# Diabetes associated with dominant insulin gene mutations: outcome of 24-month, sensor-augmented insulin pump treatment

**DOI:** 10.1007/s00592-015-0793-1

**Published:** 2015-08-04

**Authors:** Federica Ortolani, Elvira Piccinno, Valeria Grasso, Francesco Papadia, Rossana Panzeca, Claudio Cortese, Barbara Felappi, Albina Tummolo, Marcella Vendemiale, Fabrizio Barbetti

**Affiliations:** Department of Metabolic Diseases, Clinical Genetics and Diabetology, Giovanni XXIII Children’s Hospital, Via Amendola 207, 70126 Bari, Italy; Department of Experimental Medicine and Surgery, University of Tor Vergata, Via Montpellier 1, 00133 Rome, Italy; Pediatrics Unit and Neonatal Intensive Care, Valduce Hospital, 22100 Como, Italy; Department of Pediatrics, University of Brescia, 25123 Brescia, Italy; Clinical Psychology, Giovanni XXIII Children’s Hospital, 70126 Bari, Italy; Bambino Gesù Children’s Hospital, IRCCS, 00164 Rome, Italy

Insulin gene mutations, either dominant or recessive, can cause permanent neonatal diabetes mellitus (*INS*/PNDM), which is defined as diabetes with onset within 6 months of birth [1, 2]. More rarely, *INS* dominant mutations give rise to diabetes that presents during infancy [3]. In most patients with heterozygous, dominant *INS* mutations, C-peptide is in the normal-low range at diabetes outset, but over time declines to undetectable levels as a consequence of ongoing apoptosis of the pancreatic beta cells. This process is triggered by the sustained endoplasmic reticulum (ER) stress [2] induced by misfolding of mutant insulin trapped inside the beta cell. Thus, at least in principle, strategies aimed at relieving ER stress of beta cells may help to preserve endogenous insulin production from the normal allele.

During the last 8 years, we identified a total of 16 cases with dominant *INS* gene mutations from 13 different Italian centers [2–4]. Thirteen were diagnosed with *INS*/PNDM (or with diabetes with onset during infancy) (2–4 and FB unpublished observations) before year 2010 and were treated with standard insulin therapy. Currently, all of them show undetectable C-peptide values. Instead, the three patients diagnosed with a dominant *INS* mutation after year 2010 were started on continuous subcutaneous insulin infusion (CSII) within a week after diagnosis. The three patients carried the novel INS mutation c.149A>G, p.Tyr50Cys (patient 1) and the already described c.94G>A p.Gly32Ser (patient 2) and c.314T>C p.Leu105Pro (patient 3). Diabetes onset was at 1 month of age for patient 1, 7 months for patient 2, and 5 months and a half for patient 3.

In patient 1, CSII was integrated with continuous (i.e., 24 h a day) glucose monitoring (CGM). CGM was set with alarms for hypoglycemia, with a 2-h automatic interruption of insulin delivery by the pump if glucose sensor detected a value ≤4.44 mmol/l (80 mg/dl), and for hyperglycemia, with increased insulin infusion over basal level (“correction factor”) of 0.025 U for every 5.55 mmol/l (100 mg/dl) above the glucose value of 8.33 mmol/l (150 mg/dl), in order to obtain the narrowest plasma glucose fluctuations possible.

Patient 1 was discharged from the hospital after 1 month, patient 2 after 18 days, and patient 3 after 12 days. Informed consent was obtained from parents of patients. C-peptide was evaluated in patients 1 and 2 by two-site chemiluminescent immunometric assay. Assay employed for patient 1 (Liaison C-peptide, Diasorin, Saluggia, Italy) has a reported limit of detection of 0.01 ng/ml (0.003 nmol/l) and functional sensitivity of 0.03 ng/ml (0.01 nmol/l); for patient 2, the assay (Immulite 2000 C-Peptide, Siemens) has a reported limit of detection and functional sensitivity of 0.08 ng/ml (0.03 nmol/l). Blood samples for C-peptide determination were drawn after an overnight fast, without suspension of basal insulin infusion.

At follow-up as outpatients, no allergic reaction, lipo-atrophy, or -hypertrophy at the site of catheter insertion were observed; in addition, no episodes of ketosis or severe hypoglycemia requiring new hospitalization were reported for any patient.

HbA1c is not a reliable method to evaluate metabolic control below the age of 6 months, because of the interfering effect of fetal hemoglobin: consequently, we do not report on patient 1’s glycated hemoglobin during the first semester of CSII. HbA1c values determined in patients 1 and 2 six months after diabetes onset were nevertheless comparable: 50 mmol/mol (6.7 %) for patient 1 (age 7 months) and 48 mmol/mol (6.5 %) for patient 2 (age 13 months) (Table 1). At follow-up, HbA1c values 12, 18, and 24 months after diabetes onset were declining in patient 1, while patient 2 showed a more irregular pattern (Table 1). Of note, insulin dose used for patient 2 was much lower for the entire period of CSII treatment **(**Table 1**)**, suggesting that patient 2 may be more insulin-sensitive than patient 1. At strong variance with patients 1 and 2, patient 3 showed very high HbA1c from diabetes onset and never reached optimal metabolic control (Table 1).

**Table 1:** Insulin dose and HbA1c in the three patients during 24-month period of treatment with CSII

Time after diabetes onset	Patient 1				Patient 2				Patient 3			
	Total insulin (U/kg/day)	Basal (%)	Bolus (%)	HbA1c [mmol/mol (%)]	Total insulin (U/kg/day)	Basal (%)	Bolus (%)	HbA1c [mmol/mol (%)]	Total insulin (U/kg/day)	Basal (%)	Bolus (%)	HbA1c [mmol/mol (%)]
At onset	0.7	88	12	n.r.	0.15	100		53 (7)	0.7	100	0	126.2 (13.6)
6–7 months	0.57	82	18	50 (6.7)	0.15	93.2	6.8	50 (6.7)	0.46	93	7	86 (10)
12 months	0.69	85	15	43 (6.1)	0.36	85	15	39 (5.7)	0.71	93	7	69 (8.5)
18 months	0.67	89	11	44 (6.2)	0.36	87	13	38 (5.6)	0.64	93	7	69 (8.5)
24 months	0.7	87	13	39 (5.7)	0.18	87	13	54 (7.1)				

*n.r.* not reported (HbA1c determined, but value not included in the table)

**Fig. 1:**
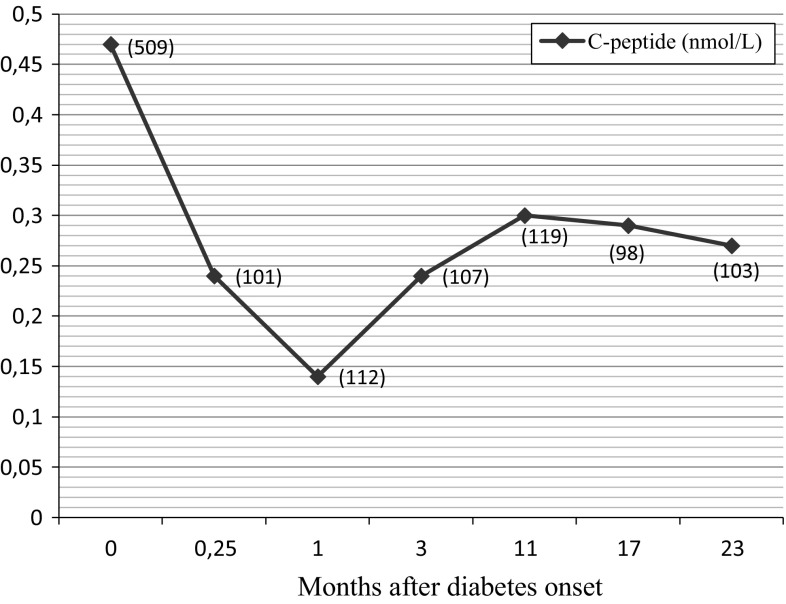
C-peptide plasma level of patient 1 during the 24-month period of integrated CSII–CGM system. *Numbers below each point* are glucose values (mg/dl) at the time of blood drawing

Interestingly, C-peptide was measurable at diabetes onset (1.04 ng/ml or 0.35 nmol/l), but became undetectable in patient 2 (CSII only) after 12 and 24 months from diabetes diagnosis, consistent with other reports showing this pattern in patients with *INS* proteotoxic mutations [2, 3]. In contrast, after the initial decrement recorded the first month after diabetes presentation, C-peptide of patient 1 (integrated system CSII/CGM) increased with a current value at 24 months of age of 0.26 nmol/l (Fig. 1). However, it has to be noticed that because of different sensitivities between C-peptide assays utilized, a direct comparison of these two patients cannot be easily made. Nevertheless, this was a surprising finding that has no easy explanation. From the clinical standpoint, the effect of the p.Tyr50Cys mutation seemed more pronounced than p.Gly32Ser, because patient 1 had an earlier diabetes presentation combined with a higher plasma glucose at onset (509 vs 293 mg/dl of patient 2). Interestingly, five patients previously described who bear *INS*/G23S mutation had diabetes onset at 6 months of age or beyond [1, 3]. On the other side of the token, patient 1 had no HbA1c fluctuations (Table 1), and it is tempting to speculate that the reduction in patient’s glycemic excursions obtained with CSII/CGM integrated system may have led to a substantial reduction in beta cell apoptosis consequent to the alleviation of endoplasmic reticulum crowding [5].

The results presented here indicate that CSII alone as well as sensor-augmented CSII is a feasible—and safe—therapeutic strategy for neonates or infants with diabetes, not only in the hospital setting, but at home. Moreover, CSII–CGM integrated system may be superior to CSII only, though we acknowledge that more cases must be studied to draw any robust conclusion. This may be probably obtained only through an international collaborative study, taking into consideration that PNDM is a rare condition (1:200,000 live births in countries with low rate of consanguineous marriages) [4].
